# Long‐term insulin resistance is associated with frailty, frailty progression, and cardiovascular disease

**DOI:** 10.1002/jcsm.13516

**Published:** 2024-06-20

**Authors:** Zezhi Ke, Han Wen, Rihua Huang, Xinghao Xu, Kevin Yang, Wenbin Liu, Suisui Wang, Xu Zhang, Ye Guo, Xinxue Liao, Xiaodong Zhuang, Jie Zhao, Litao Pan, Lizhen Liao

**Affiliations:** ^1^ School of Health Science Guangdong Pharmaceutical University Guangzhou P. R. China; ^2^ Guangdong Key Laboratory of Bioactive Drug Research Guangdong Pharmaceutical University Guangzhou P. R. China; ^3^ Department of Cardiology The First Affiliated Hospital of Sun Yat‐Sen University Guangzhou P. R. China; ^4^ NHC Key Laboratory of Assisted Circulation Guangzhou P. R. China; ^5^ Department of Acupuncture and Massage Shenzhen Second People's Hospital Shenzhen P. R. China

**Keywords:** Cardiovascular disease, Frailty, HOMA‐IR trajectory, Insulin resistance

## Abstract

**Background:**

Insulin resistance and diabetes are associated with an increased risk of frailty, and frailty is associated with cardiovascular disease and premature mortality. We aim to investigate the impact of long‐term insulin resistance trajectories on future frailty and cardiovascular risk among young adults.

**Methods:**

In total, 3168 participants with a 30‐year follow‐up period. The baseline period covered the first 15 years as the exposure period. Insulin resistance was determined using the homeostasis model assessment for insulin resistance (HOMA‐IR), and three trajectories (low, moderate, and high) were constructed. The subsequent 15 years constituted the event accrual period. Frailty was assessed using a deficit accumulation mode, and cardiovascular outcomes were obtained from the 15‐year event accrual period.

**Results:**

The mean age of all 3168 participants was 41.0 (37.0–43.0) years, with 1750 (55.2%) being women. Participants in the high level of insulin resistance trajectory had an increased prevalence of frailty (OR: 1.55, 95% CI: 1.05–2.30, *P* = 0.028). Although no statistically significant associations were observed after full adjustment, single‐factor analysis indicated association between the moderate and high trajectories and frailty progression. Additionally, participants with high level of insulin resistance trajectory were associated with an increased risk of cardiovascular disease, coronary heart disease, and stroke. A notable correlation between HOMA‐IR trajectory and cardiovascular diseases was still discernible within the subgroup where the frailty index ≥0.12 (HR: 2.12, 95% CI: 1.17–3.83, *P* = 0.013) (*P* for interaction >0.05).

**Conclusions:**

Long‐term high level of insulin resistance is associated with high prevalence of frailty, and an increased risk of cardiovascular events. Emphasizing the importance of early prevention and intervention for abnormal glucose metabolism in young adults to prevent frailty and cardiovascular disease.

## Introduction

Frailty is a physiological decline in function that occurs with age, such as physical and cognitive decline, reduced physical activity, and more. Its hallmark is the increased vulnerability of physiological processes to various risk and stress factors, particularly pronounced in the elderly.^1^ Recent research suggested that frailty is not confined to old age and occurs continuously, with young adults accumulating frailty risks that may manifest as a more pronounced frailty trajectory than their peers when they enter middle and old age.^2^ Early identification and intervention of weakness in young adults may yield greater benefits compared with strategies solely targeting frail elderly individuals. The frailty index (FI) is an indicator used to calculate the biological age of a population, and its association with mortality and cardiovascular events is commonly reported.^3^

The pathological and physiological mechanisms of aging, such as oxidative stress, inflammation, as well as declining muscle function, have been observed in insulin resistance populations.^4^, ^5^, ^6^ Cohort studies have shown an association between metabolic syndrome or insulin resistance and an increased risk of frailty.^7^, ^8^, ^9^ However, most frailty related research has been conducted solely in older adults, and previous literatures has primarily focused on the impact of baseline insulin resistance on frailty, neglecting the effects of long‐term changes in insulin resistance on frailty and outcomes.^8^ Therefore, it is necessary to investigate the longitudinal patterns of insulin resistance in relation to frailty and cardiovascular diseases to understand its long‐term contributions to physiological aging. It has been demonstrated that long‐term HOMA‐IR trajectories are associated with cardiovascular disease incidence and mortality^10^ and that trajectories of triglyceride‐glucose index may also help to identify older people at higher risk,^11^ but there is a lack of evidence for the relevance of these in younger populations.

Here, we quantified insulin resistance using the homeostasis model assessment for insulin resistance (HOMA‐IR) and constructed a long‐term HOMA‐IR time series. Frailty was quantified using the FI. The primary aim of this research is to explore the predictive value of long‐term HOMA‐IR trajectories in assessing frailty status and its progression in young individuals, as well as to investigate their association with cardiovascular diseases.

## Methods

This cohort study was approved by the institutional review boards of the central institutions in each field. All participants were informed and provided written informed consent. This study strictly followed the Strengthening the Reporting of Observational Studies in Epidemiology (STROBE) guidelines for reporting cohort studies.

### Study design and participants

**Figure 1:**
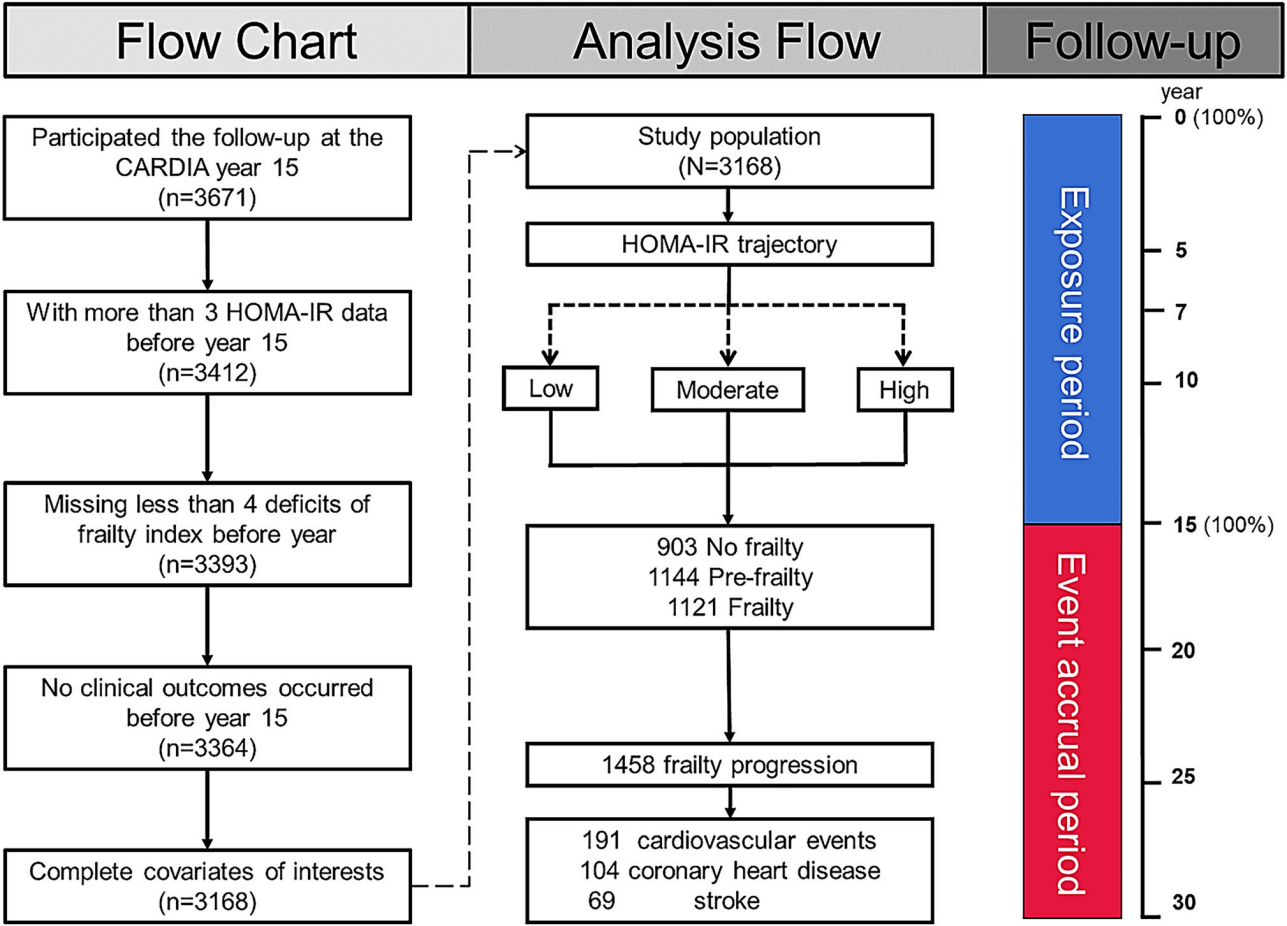
Study flow of the exposure and the event accrual periods. The 30‐year follow‐up period for study participants was divided into a 15‐year exposure period (from Y0‐Y15) and a 15‐year event accrual period (from Y15 to Y30). First, they were examined at least three times during the event‐exposure period, and second, had no more than four deficits missing and were free of cardiovascular disease, coronary artery disease, and stroke at baseline; appropriate covariates were included in the analyses. A total of 3168 participants were included in the final analysis.

The study design and participant characteristics of the Coronary Artery Risk Development in Young Adults (CARDIA) have been previously reported.^12^ Briefly, participants in the CARDIA were recruited from 5115 whites and blacks from four sites (Birmingham, Alabama; Oakland, California; Chicago, Illinois; and Minneapolis, Minnesota). Participants in this cohort had their first examination (aged 18–30 years) since 1985–1986, and they have received eight cycles of examinations (at 2, 5, 7, 10, 15, 20, 25, and 30 years after baseline, respectively) over the subsequent 30 years of follow‐up. The flow of this study is shown in Figure 1, which firstly included all participants (*N* = 3671) who had participated in CARDIA Year 15 follow‐up with available HOMA‐IR data on at least three occasions from Year 0 to Year 15 (0, 7, 10, and 15), and secondly had no more than four missing FI constituent entries, and no prior 15‐year cardiovascular disease outcomes. The final analytic sample consisted of 3168 CARDIA participants.

### Insulin resistance trajectory

**Figure 2:**
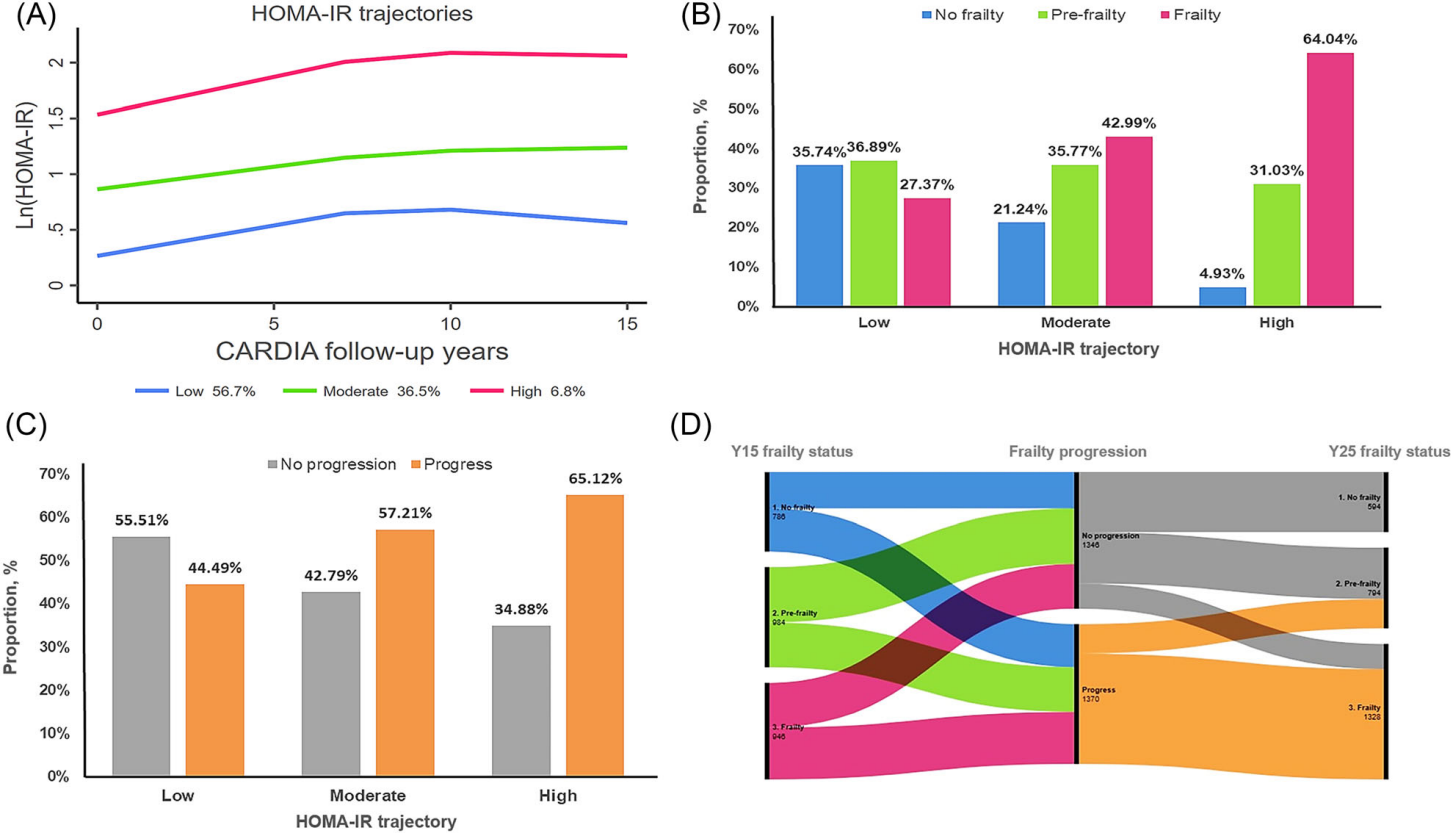
HOMA‐IR trajectory of the year 0–15 follow‐up in CARDIA (A). Distribution of frailty status in different HOMA‐IR trajectory (B). Distribution of frailty progression in different HOMA‐IR trajectory (C), and Sankey diagram of frailty progression in a decade in CARDIA (D).

HOMA‐IR was calculated using the following equation: fasting glucose (mg/dL)*fasting insulin (μU/dL)/405.^13^ Because of the methodological requirement for data normalization, we converted all HOMA‐IR data to natural logarithms before constructing the trajectory model, and subsequently used a group‐based trajectory model to create a 0–15 year HOMA‐IR trajectory model. Using the HOMA‐IR trajectory model helped us identify groups of participants who had similar changes in HOMA‐IR levels from youth to middle age.^14^ The shape of the trajectories (linear, squared, or cubic) and the number of groups were evaluated by the Bayesian information criterion, and the mean posterior probability was used to assess this, respectively (Table S1), and eventually, the classification of all participants into three groups was the most appropriate, with the HOMA‐IR trajectories classified into three groups, low (1.8 [1.4–2.3]), moderate (3.5 [2.8–4.7]), and high (8.1 [5.7–11.7]), respectively (Figure 2A).

### Frailty index and progression of frailty

The construction of the FI was carried out in strict accordance with standard procedures^15^ and deficits included in the FI should meet the following criteria: The deficits are health‐related and increase in risk with age but are not age‐related traits; the deficits involve multiple body systems and a range of physiological domains; the deficits do not saturate middle‐aged adults; and the deficits do not have more than a 5% missingness rate in the CARDIA population. Finally, we chose 31 variables for the calculation of the FI (Table S2), variables included self‐reported baseline condition, physician diagnosis or self‐reported medical condition (physical measurements, symptoms, and signs), and the quality of life questionnaire SF‐12. All deficits were assigned a score in the range 0.00–1.00, with 0.00 indicating no deficits (a state of relatively good health) and 1.00 indicates the presence of a deficit or a significant deficit (a relatively unhealthy state).^16^ FI was calculated by dividing the total deficit score for each individual by 31 (or by the total number of deficits held by participants with three or fewer deficits missing). FI is a continuous variable ranging from 0.00–1.00, with larger values indicating a more significant state of frailty. In order to better group all participants, we further categorized into three frailty states: No Frailty (FI < 0.12), Pre‐frailty (0.12 ≤ FI ≤ 0.20) and Frailty (FI > 0.20). The cut‐off points of 0.12 and 0.20 roughly corresponded to three and six deficiencies, respectively.

Similarly, we constructed FI with the same deficits in the year 25 of follow‐up data. Frailty progression was assessed as from year 15 to year 25 of follow‐up: (1) the progression of the frailty status from No frailty to Pre‐frailty or Frailty, (2) the progression of the frailty status from Pre‐frailty to Frailty, and (3) an increase >0 FI among those with Frailty at baseline. It was ultimately determined that 1370 participants experienced frailty progression.

### Outcomes

Two physicians from the Endpoint Committee independently reviewed the medical records and assessed fatal events or morbid events according to pre‐specified standardized procedures.^14^ Detailed information such as definitions of diseases and assessment criteria have been described in the CARDIA Endpoint Events Manual of Operations.^17^ Our outcomes were derived from the year 30 exam, included any fatal‐ and non‐fatal cardiovascular disease and coronary heart disease, as well as stroke. Results for the current analysis are available through 2019.

### Statistical analysis

Baseline characteristics are represented by the three HOMA‐IR trajectories (low, medium, and high), with skewed continuous variables expressed as median (upper quartile ‐ lower quartile) or categorical variables expressed as *n* (%). The Kruskal–Wallis test or the chi‐square test were used appropriately to test for statistical differences between groups. We used clustered bar charts to visualize the different frailty status and the percentage of FI progression in three trajectories, respectively. In addition, we used Sankey diagram to visualize more clearly the distribution of the flow of FI progression in participants after a decade.

The association between HOMA‐IR trajectory and frailty was generalized by logistic regression analyses yielding an advantage ratio (OR) and 95% confidence interval (95% CI). We constructed logistic models with asymptotic correction for two covariates, model 1 adjusted for age, gender, and ethnicity. Model 2 was further adjusted for BMI, SBP, DBP, smoking, drinking, LDL‐c, HDL‐c, CRP, and diabetes at baseline.

Hazard ratios (HRs) and 95% CI for the associations between HOMA‐IR trajectories and cardiovascular events, coronary heart disease, and stroke were estimated using Cox proportional hazards regression models, again applying the above models to adjust for confounders. All categorical variables were converted to continuous variables for linear trend tests and included in Cox proportional hazards regression models. The Kaplan–Meier method was used to calculate the cumulative incidence of cardiovascular events, coronary heart disease, and stroke in different HOMA‐IR trajectory subgroups, and log‐rank test was used to compare differences between groups.

Statistical analyses were performed in Stata version 17.0 (Stata Corp LLC, Texas, USA) and SPSS version 26 (SPSS, Chicago, IL), and a two‐sided *P* < 0.05 was considered statistically significant.

## Results

### Baseline characteristics

The distribution of the frailty status in different HOMA‐IR trajectories is shown in Figure 2B. In general, the clustered bar graphs indicate that the prevalence of frailty increases as the level of the HOMA‐IR trajectory rises. In addition, the absence of frailty was more prominent at lower HOMA‐IR levels, while pre‐frailty was more evenly distributed across intensity levels. Rather, frailty was significantly increased at higher levels of insulin resistance.

In the cohort as a whole (mean age 41.0 years, 55.2% female, 45.8% black), 71% of participants had a FI > 0.12. Table 1 shows baseline characteristics of participants stratified by different HOMA‐IR trajectories. Participants did not have statistical different by age, sex, smoking, or serum creatinine. Among those with higher HOMA‐IR levels, they had higher body mass index and systolic blood pressure, larger abdominal circumference, less physical activity, and a higher prevalence of comorbidities, with the diabetes population being more prevalent among participants with high HOMA‐IR levels (2.4% in low and 33.5 in high, *P* < 0.001). In addition, rates of other medication use were higher in high HOMA‐IR trajectory level. Table S3 shows baseline characteristics categorized according to frailty status; Table S4 shows baseline characteristics comparing individuals included and excluded from the study cohort.

**Table 1:** Participant characteristics by HOMA‐IR trajectory

	Total (*n* = 3168)	HOMA‐IR trajectory			*P* value
		Low (*n* = 1816)	Moderate (*n* = 1149)	High (*n* = 204)	
HOMA‐IR	2.4 (1.7–3.6)	1.8 (1.4–2.3)	3.5 (2.8–4.7)	8.1 (5.7–11.7)	<0.001
Age, years	41.0 (37.0–43.0)	41.0 (38.0–43.0)	40.0 (37.0–43.0)	41.0 (37.3–44.0)	0.137
Women, *n* (%)	1750 (55.2)	1008 (55.5)	638 (55.5)	104 (51.2)	0.494
Black, *n* (%)	1450 (45.8)	654 (36.0)	659 (57.4)	137 (67.5)	<0.001
SBP, mmHg	110.0 (103.0–120.0)	108.0 (101.0–116.0)	114.0 (106.0–124.0)	119.0 (110.0–132.0)	<0.001
DBP, mmHg	73.0 (67.0–81.0)	71.0 (65.0–78.0)	76.0 (69.0–83.0)	79.0 (72.0–88.0)	<0.001
BMI, kg/m^2^	27.2 (23.9–32.0)	25.0 (22.6–27.7)	31.0 (27.4–35.6)	37.1 (32.5–41.1)	<0.001
Waist circumstance, cm	87.5 (78.0–98.0)	81.5 (74.0–89.0)	96.0 (88.0–104.0)	111.0 (101.5–120.0)	<0.001
Total physical activity, EU	286.0 (143.0–492.8)	324.0 (167.0–542.8)	240.0 (107.0–437.5)	177.0 (72.0–348.0)	<0.001
Smoking, *n* (%)	665 (21.0)	392 (21.6)	241 (21.0)	32 (15.8)	0.136
Drinking, *n* (%)	2516 (79.5)	1524 (83.9)	856 (74.5)	138 (68.0)	<0.001
Fasting glucose, mg/dL	83.0 (78.0–90.0)	81 (76.0–86.0)	86 (81.0–92.0)	98 (88.0–122.0)	<0.001
Fasting insulin, uU/mL	12.0 (8.0–17.0)	9.0 (7.0–11.0)	17.0 (13.0–21.0)	31.0 (22.0–41.0)	<0.001
TC, mg/dL	181.0 (160.0–204.8)	179.0 (158.0–202.0)	184.0 (165.0–209.5)	178.0 (159.0–203.0)	<0.001
TG, mg/dL	82.0 (59.0–119.0)	72.0 (53.0–99.8)	95.0 (68.0–140.0)	115.0 (83.5–156.0)	<0.001
HDL‐c, mg/dL	49.0 (40.0–59.0)	52.0 (44.0–63.0)	45.0 (38.0–54.0)	40.0 (34.0–47.0)	<0.001
LDL‐c, mg/dL	110.0 (91.0–133.0)	107.0 (87.0–129.0)	116.0 (97.0–138.0)	112.0 (93.3–133.0)	<0.001
VLDL, mg/dL	16.0 (12.0–24.0)	14.0 (11.0–20.0)	19.0 (14.0–28.0)	23.0 (17.0–31.0)	<0.001
Cr, mg/dL	1.0 (0.9–1.1)	1.0 (0.9–1.1)	1.0 (0.9–1.1)	1.0 (0.9–1.1)	0.337
CRP, μg/mL	1.1 (0.8–2.2)	1.0 (0.8–1.5)	1.5 (1.0–3.0)	2.6 (1.2–4.4)	<0.001
Hypertension, *n* (%)	483 (15.2)	162 (8.9)	235 (20.5)	86 (42.2)	<0.001
Diabetes, *n* (%)	166 (5.2)	43 (2.4)	55 (4.8)	68 (33.5)	<0.001
Hyperlipidaemia, *n* (%)	548 (17.3)	263 (14.5)	238 (20.7)	47 (23.2)	<0.001
Medication for hypertension, *n* (%)	215 (6.8)	42 (2.3)	116 (10.1)	57 (28.1)	<0.001
Lipid‐lowering therapy, *n* (%)	62 (2.0)	21 (1.2)	26 (2.3)	15 (7.4)	<0.001
Aspirin, *n* (%)	172 (5.4)	87 (4.8)	66 (5.7)	19 (9.4)	0.021

### Impact of homeostasis model assessment for insulin resistance trajectories for frailty and frailty progression

For frailty status, among different HOMA‐IR trajectories, participants with moderate and high HOMA‐IR trajectories showed a higher prevalence of frailty compared with participants without frailty [moderate (OR: 2.04, 95% CI: 1.78–2.34, *P* < 0.001) and high (OR: 2.04, 95% CI: 3.86–7.02, *P* < 0.001)]. After fully adjusted, participants with high HOMA‐IR trajectory continued to show an associated with frailty severity increased (OR: 1.55, 95% CI: 1.05–2.30, *P* = 0.028) (Table 2). Regarding frailty progression, we initially observed an association in the univariate analysis among participants with moderate or high insulin resistance trajectories. However, after thorough adjustments in the model, no significant correlation was identified.

**Table 2:** Relationship between HOMA‐IR trajectories and frailty status and frailty progression

HOMA‐IR trajectory	Unadjusted		Model 1		Model 2	
	OR (95% CI)	*P* value	OR (95% CI)	*P* value	OR (95% CI)	*P* value
Frailty status						
Low	Reference	‐	Reference	‐	Reference	‐
Moderate	2.04 (1.78–2.34)	<0.001	2.06 (1.78–2.38)	<0.001	1.18 (0.99–1.40)	0.067
High	5.20 (3.86–7.02)	<0.001	5.81 (4.24–7.95)	<0.001	1.55 (1.05–2.30)	0.028
*P* for trend	‐	<0.001	‐	<0.001	‐	0.015
Frailty progression						
Low	Reference	‐	Reference	‐	Reference	‐
Moderate	1.64 (1.40–1.92)	<0.001	1.61 (1.37–1.89)	<0.001	0.90 (0.62–1.30)	0.115
High	2.37 (1.73–3.26)	<0.001	2.27 (1.64–3.13)	<0.001	0.73 (0.49–1.08)	0.563
*P* for trend	‐	<0.001	‐	<0.001		0.023

Model 1: Adjust for age, sex, and race. Model 2: Adjust for model 1 plus BMI, smoking, drinking, LDL‐c, CRP, hypertension, diabetes, hyperlipidaemia, lipid‐lowering therapy, and aspirin.

During follow‐up, the proportion of participants who had FI progression over a decade was lower among those with low HOMA‐IR trajectory (44.49%). The proportion of participants who had FI progression was higher than the proportion of those who did not had frailty in both the moderate and high HOMA‐IR trajectories. The highest proportion of participants who had FI progression among those with the high HOMA‐IR trajectory (65.12%) (Figure 2C). The Sankey diagram visualizing the progression of frailty is shown in Figure 2D, in terms of different FI progression for different frailty status at baseline, with those who were pre‐frailty and frailty undergoing more portion of progression after a decade, with more people progressing to frailty.

### Association between homeostasis model assessment for insulin resistance trajectory with cardiovascular disease, coronary heart disease, and stroke

During the event accrual period, cardiovascular events occurred in 191 individuals, coronary heart disease in 104 individuals, and stroke in 69 individuals, respectively. The cumulative incidence of all outcomes (cardiovascular incident, coronary heart disease, and stroke) was highest in the high HOMA‐IR trajectory group, followed by the moderate HOMA‐IR trajectory group, and the low HOMA‐IR trajectory group (Figure S1). Table 3 displays the Cox proportional hazards analysis between the HOMA‐IR trajectory and each event separately. The single‐factor Cox regression model revealed that both moderate and high HOMA‐IR trajectories were associated with increased risks of cardiovascular disease, coronary heart disease, and stroke. After fully adjusting for potential confounding factors, elevated risks were consistently observed for all events within the high HOMA‐IR trajectory [cardiovascular disease (HR: 2.62, 95% CI: 1.49–4.61, *P* = 0.001), coronary heart disease (HR: 3.02, 95% CI: 1.34–6.79, *P* = 0.008), and stroke (HR: 3.37, 95% CI: 1.39–8.20, *P* = 0.007)]. Linear trend tests for all outcomes across all models yielded results with *P*‐values <0.05. Although the *P* for interaction was found to be greater than 0.05 in our endeavour to investigate the interaction between insulin resistance and frailty concerning cardiovascular outcomes, a notable correlation between HOMA‐IR trajectory and cardiovascular diseases was still significant within the subgroup where the FI ≥ 0.12 (HR: 2.12, 95% CI: 1.17–3.83, *P* = 0.013) (Table 4).

**Table 3:** Incident outcomes by HOMA‐IR trajectory

HOMA‐IR trajectory	*n* (%)	Unadjusted		Model 1		Model 2	
		HR (95% CI)	*P* value	HR (95% CI)	*P* value	HR (95% CI)	*P* value
Cardiovascular disease							
Low	72 (4.0)	Reference		Reference		Reference	
Moderate	81 (7.0)	1.82 (1.33–2.50)	<0.001	1.70 (1.24–2.35)	0.001	1.34 (0.93–1.93)	0.114
High	38 (18.7)	5.16 (3.48–7.61)	<0.001	4.39 (2.94–6.57)	<0.001	2.62 (1.49–4.61)	0.001
*P* for trend			<0.001		<0.001		0.002
Coronary heart disease							
Low	42 (2.3)	Reference		Reference		Reference	
Moderate	44 (3.8)	1.68 (1.10–2.57)	0.016	1.76 (1.14–2.69)	0.010	1.53 (0.95–2.47)	0.080
High	18 (8.9)	4.00 (2.31–6.96)	<0.001	3.94 (2.24–6.92)	<0.001	3.02 (1.34–6.79)	0.008
*P* for trend			<0.001		<0.001		0.010
Stroke							
Low	21 (1.2)	Reference		Reference		Reference	
Moderate	29 (2.5)	2.22 (1.26–3.89)	0.005	1.76 (0.99–3.11)	0.052	1.51 (0.80–2.87)	0.206
High	19 (9.4)	8.59 (4.62–15.97)	<0.001	6.14 (3.25–11.60)	<0.001	3.37 (1.39–8.20)	0.007
*P* for trend			<0.001		<0.001		0.010

Model 1: Adjust for age, sex, and race. Model 2: Adjust for model 1 plus BMI, smoking, drinking, LDL‐c, CRP, hypertension, diabetes, hyperlipidaemia, lipid‐lowering therapy, and aspirin.

**Table 4:** Interactions between HOMA‐IR trajectories and FI status

HOMA‐IR trajectory, FI status*	*n* (%)	Unadjusted		Model 1		Model 2	
		HR (95% CI)	*P* value	HR (95% CI)	*P* value	HR (95% CI)	*P* value
Low, FI < 0.12	17 (2.6)	Reference	‐	Reference	‐	Reference	‐
Moderate and High, FI < 0.12	12 (4.7)	1.83 (0.87–3.82)	0.110	1.62 (0.77–3.39)	0.203	1.47 (0.69–3.11)	0.319
Low, FI ≥ 0.12	55 (4.7)	1.83 (1.07–3.16)	0.029	2.26 (1.31–3.92)	0.004	1.58 (0.90–2.78)	0.115
Moderate and High, FI ≥ 0.12	107 (9.7)	3.93 (2.36–6.56)	<0.001	4.33 (2.57–7.30)	<0.001	2.12 (1.17–3.83)	0.013

Model 1: Adjust for age, sex, and race. Model 2: Adjust for model 1 plus BMI, smoking, drinking, LDL‐c, CRP, hypertension, diabetes, hyperlipidaemia, lipid‐lowering therapy, and aspirin.

* Multiplicative interaction: *P* for interaction >0.05.

## Discussion

In this prospective cohort of 3168 young adults followed for 30 years, we categorized eligible participants into subgroups with different time‐series insulin resistance trajectories (low, moderate, and high), in addition to constructing a frailty index, which included various risk factors, disease histories, and scales. We found that, long‐term high‐level insulin resistance was associated with a worse future frailty status, as specified by the fact that participants with a long period of high HOMA‐IR had a worse risk of frailty status. Simultaneously, participants with prolonged high insulin resistance trajectories exhibited a higher risk of cardiovascular disease, coronary heart disease, and stroke. Regarding the frailty progression, while the association with long‐term insulin resistance was not evident after full adjusted, significant associations were still observed in univariate analyses; moreover, participants who were on moderate and high HOMA‐IR trajectories at baseline experienced more future frailty progression. Furthermore, despite the absence of an interaction between insulin resistance trajectories and frailty status, we can still observe that in participants with pre‐frailty and frailty, trajectory for moderate and high insulin resistance is associated with high risk of cardiovascular disease.

This study demonstrates that individuals with prolonged higher levels of insulin resistance, compared with those with low insulin resistance level, are associated with a higher susceptibility to frailty. Compared with previous studies, there are similarities and differences. Several studies indicate a relationship between insulin resistance and frailty, with one of the relevant mechanisms being the muscular dysfunction (often observed as one of the components of frailty syndrome) resulting from the metabolic disturbances characteristic of insulin resistance, characterized by a decline in muscle mass and loss of strength.^8^, ^9^, ^18^ However, distinctions arise as follows: Firstly, to our knowledge, our study is the first to reveal the association between long‐term insulin resistance and the frailty status, and further, frailty progression. Compared with baseline analyses that only provide a single time point, trajectory modelling offers a longitudinal perspective that captures dynamic changes in insulin resistance status. By capturing longitudinal changes and individual variability, trajectory analysis enhances the ability to predict future health outcomes and tailor intervention strategies accordingly. Secondly, whereas previous studies primarily focused on frailty in the elderly,^6^ our study targeted young adults, emphasizing the importance of early attention and intervention regarding the impact of metabolic abnormalities on frailty and cardiovascular diseases. Lastly, past research mainly centred on metabolic syndrome rather than the relationship between insulin resistance and frailty. Insulin resistance constitutes a core component and physiologic determinant of metabolic syndrome,^19^ as evidenced by a meta‐analysis concerning the interplay between metabolic syndrome and frailty, demonstrating a more prevalent frailty among elderly individuals with metabolic syndrome.^20^ Central obesity is a crucial factor contributing to insulin resistance in frail elderly individuals,^21^ cross‐sectional studies have also suggested correlations between different components of metabolic syndrome and frailty, such as abdominal obesity, elevated triglycerides, reduced high‐density lipoprotein cholesterol, hyperglycaemia, and hypertension.^9^ Conversely, previous investigations have demonstrated no substantial link between metabolic syndrome and frailty. In contrast, frailty has been associated with insulin resistance and inflammation, hypothesized as a high‐risk factor for frailty and disability in the elderly, primarily attributed to glucose dysregulation.^18^ A similar study focused on frailty progression in diabetic patients found that individuals without frailty at baseline displayed a stronger association with a higher FI due to elevated late‐life HbA1c levels, along with increased diabetes prevalence.^22^

Addressing the underlying risk factors for frailty has the potential to delay or even prevent the onset of frailty, which, in turn, serves as a precursor to cardiovascular disease outcomes.^23^ Our study also found that insulin resistance long‐term time series was associated with cardiovascular events, coronary heart disease, and stroke, consistent with the results of a recent study.^10^ Our study did not reveal a relationship between insulin resistance trajectories and frailty; these two factors appear to be non‐synergistic. Consequently, the correlation between insulin resistance and cardiovascular disease remains unaltered by frailty status, and vice versa. Several potential mechanisms are thought to underlie this connection. First, insulin resistance can trigger lipid abnormalities, such as elevated triglycerides and reduced high‐density lipoprotein cholesterol, contributing to the development of atherosclerosis. Second, insulin resistance is strongly correlated with chronic low‐grade inflammation, oxidative stress, and impaired endothelial function, all of which can accelerate the progression of cardiovascular diseases.^24^

Our study carries several crucial clinical implications. Firstly, it uncovers the elevated risks of frailty and cardiovascular disease in young to middle‐aged adults who persistently maintain high levels of insulin resistance. This emphasizes the significance of early detection and intervention in metabolic irregularities among adults, serving as a preventive measure against both physiological aging and cardiovascular events. Secondly, considering the HOMA‐IR as a substitute marker for the gold standard clamp technique used to measure insulin resistance, in conjunction with trajectory analysis, serves to quantitatively gauge the extent of prolonged insulin resistance among young adults. This integration empowers clinical practitioners with the capability to promptly identify metabolic states in patients, thereby furnishing substantial support for the formulation of individualized therapeutic strategies and health recommendations. Thirdly, in line with the American Diabetes Association's ‘Standards of Medical Care in Diabetes’ released in 2022,^25^ in the context of young adults with disrupted glucose metabolism, clinicians are encouraged to accentuate early interventions. Fourth, results highlighting the importance of incorporating medication treatment patterns into the analysis for a more holistic picture of outcomes. These interventions encompass tailored health education initiatives targeting individuals displaying elevated levels of insulin resistance. The comprehensive approach involves judicious pharmaceutical interventions, appropriate levels of physical activity, and cultivation of healthy lifestyle practices. This strategic approach not only mitigates the risk of frailty progression but also curbs the potential for cardiovascular events.

There are several limitations existed. Firstly, we defined frailty using a deficit accumulation model, in reality, constructing a FI within the CARDIA dataset posed significant challenges due to factors such as loss to follow‐up and the absence of certain critical variables in FI construction. Furthermore, the subjectivity involved in constructing the FI highlights the necessity for more standardized procedures in future research. Secondly, the specific thresholds we employed to categorize pre‐frailty (FI ≥ 0.12) and frailty (FI > 0.20) were derived from estimates of the number of deficits, yet these thresholds have not been independently validated in other clinical datasets. This lack of validation in similar datasets raises a concern. Thirdly, our study population consists of young adults, with relatively low occurrences of outcomes, particularly cardiovascular diseases. The numbers within each subgroup are even fewer. This limited occurrence poses challenges in fully exploring the interaction between frailty and HOMA‐IR trajectory as well as their correlation with outcomes. Fourthly, our study maintains an observational design, which restricts the establishment of causal relationships. Furthermore, we acknowledge that residual confounding factors were not completely addressed in our analyses.

## Conclusions

In conclusion, this analysis represents the first to reveal the association between long‐term insulin resistance and the increased risk of future frailty and cardiovascular events. Our findings expand the understanding of the adverse consequences of abnormal glucose metabolism in young adults, emphasizing the necessity to identify and intervene on insulin resistance at an early stage.

## Funding

This study was supported by the Shenzhen Science and Technology Program (JCYJ20230807115308018).

## Conflict of interest

All the authors declared no conflicting interests with respect to the research, authorship, or publication of this article.

## Supporting information


**Table S1.** The statistics for trajectory models for HOMA‐IR.
**Table S2.** The defects constituting the frailty index.
**Table S3.** Participant Characteristics by frailty status.
**Table S4.** Participant Characteristics by frailty status.
**Figure S1.** Cumulative incidence of cardiovascular disease (A), coronary heart disease (B), and stroke (C) by HOMA‐IR trajectory.

## Data Availability

All CARDIA data are obtained from the CARDIA Coordinating Center (https://www.cardia.dopm.uab.edu/contact‐cardia). Details of the National Heart, Lung, and Blood Institute policies governing the data and how to access these data are available at (https://www.cardia.dopm.uab.edu/study‐information/nhlbi‐data‐repository‐data).
