# Supplementary material for: Long‐term insulin resistance is associated with frailty, frailty progression, and cardiovascular disease
Source: J Cachexia Sarcopenia Muscle. 2024 Jun 20;15(4):1578–86. doi: 10.1002/jcsm.13516 (PMC11294012; doi:10.1002/jcsm.13516)

Table S1. The statistics for trajectory models for HOMA-IR.

Trajectory groups	Trajectory shape parameter *	BIC N=3168	BIC N=12165	AIC N=3168	Group membership (%)	Avepp
2	(3 3)	-9240.34	-9247.06	-9210.03	76.46	0.963
					23.54	0.905
2	(2 2)	-9254.93	-9260.32	-9230.69	76.47	0.963
					23.53	0.904
3	(3 3 2)	-8842.67	-8852.09	-8800.25	56.69	0.915
					36.53	0.858
					6.78	0.914
3	(3 2 2)	-8844.11	-8852.85	-8804.71	57.02	0.916
					36.24	0.857
					6.74	0.916
4	(3 3 3 2)	-8742.69	-8755.47	-8685.11	45.38	0.869
					40.87	0.824
					12.11	0.846
					1.64	0.911
4	(3 2 2 2)	-8744.40	-8755.84	-8692.89	46.18	0.872
					40.24	0.822
					11.92	0.843
					1.66	0.895
5	(2 2 1 1 1)	-8757.38	-8768.81	-8705.86	36.33	0.835
					43.58	0.790
					2.45	0.756
					14.85	0.781
					2.79	0.869

BIC: Bayesian information criterion, AIC: Akaike information criterion, Avepp: Average posterior probability.

*** Defines the shape parameters of the trajectory groups: 0 = intercept only, 1 = linear, 2 = quadratic, 3 = cubic.**

Table S2. The defects constituting the frailty index.

	Item	Reported	Score
1	Body-mass index (BMI) (kg/m^2)	≥25 but <30	0.50
		<18.5 or ≥30	1.00
2	Physical activity (EU)	<300	1.00
3	Currently smoking?	Yes	1.00
4	Currently drinking?	Yes	1.00
5	C-reactive protein (ug/mL)	>5	1.00
6	Urine protein creatinine ratio (mg/g)	≥30	1.00
7	Hypertension?	Yes	1.00
8	Diabetes?	Yes	1.00
9	High cholesterol?	Yes	1.00
10	Coronary artery calcium score >0	Yes	1.00
11	Leaking urine?	Yes	1.00
12	Asthma?	Yes	1.00
13	Digestive diseases?	Yes	1.00
14	Stiff or sore joints?	Yes	1.00
15	Headaches?	Yes	1.00
16	Forgetfulness?	Yes	1.00
17	Depression?	Yes	1.00
18	CES-D score	>16 but ≤19	0.50
		≥20	1.00
19	Number of medications for diseases	1-2	0.50
		≥3	1.00
	Quality of life SF-12		
20	Q1: In general, would you say your health is?	Excellent	0.00
		Very good	
		Good	
		Fair	0.50
		Poor	1.00
The following items are about activities you might do during a typical day. Does your health now limit you in these activities? If so, how much?			
21	Q2: Moderate activities such as moving a table, pushing a vacuum cleaner, bowling, or playing golf?	Not limited	0.00
		Limited a little	0.50
		Limited a lot	1.00
22	Q3: Climbing several flights of stairs?	Not limited	0.00
		Limited a little	0.50
		Limited a lot	1.00
During the past 4 weeks, have you had any of the following problems with your work or other regular daily activities as a result of your physical health?			

23	Q4: Accomplished less than you would like?	No	0.00
		Yes	1.00
24	Q5: Were limited in the kind of work or other activities?	No	0.00
		Yes	1.00
During the past 4 weeks, have you had any of the following problems with your work or other regular daily activities as a result of any emotional problems (such as feeling depressed or anxious)?			
25	Q6: Accomplished less than you would like?	No	0.00
		Yes	1.00
26	Q7: Were limited in the kind of work or other activities?	No	0.00
		Yes	1.00
27	Q8: During the past 4 weeks, how much did pain interfere with your normal work (including work outside the home and housework)?	Not at all	0.00
		A little bit	0.25
		Moderately	0.50
		Quite a bit	0.75
		Extremely	1.00
These questions are about how you feel and how things have been with you during the past 4 weeks.			
28	Q9: Have you felt calm and peaceful?	All of the time	0.00
		Most of the time	
		A good bit of the time	
		Some of the time	
		A little of the time	0.50
		None of the time	1.00
29	Q10: Did you have a lot of energy?	All of the time	0.00
		Most of the time	
		A good bit of the time	
		Some of the time	
		A little of the time	0.50
		None of the time	1.00
30	Q11: Have you felt downhearted and blue?	None of the time	0.00
		A little of the time	
		Some of the time	0.25
		A good bit of the time	0.50
		Most of the time	0.75
		All of the time	1.00
31	Q12: During the past 4 weeks, how much of the time has your physical health or emotional problems interfered with your social activities (like visiting with friends, relatives, etc.)?	None of the time	0.00
		A little of the time	
		Some of the time	0.25
		A good bit of the time	0.50
		Most of the time	0.75
		All of the time	1.00

Table S3. Participant Characteristics by frailty status.

	`Total (n=3168)	Frailty status			P value
		No frailty (n=903)	Pre-frailty (n=1144	Frailty (n=1121)	
HOMA-IR	2.4 (1.7-3.6)	2.0 (1.5-2.9)	2.3 (1.6-3.5)	2.9 (2.0-4.6)	<0.001
Age, years	41.0 (37.0-43.0)	40.0 (37.0-43.0)	41.0 (37.0-43.0)	41.0 (38.0-44.0)	<0.001
Women, n(%)	1750 (55.2)	315 (34.9)	610 (53.3)	825 (73.6)	<0.001
Black, n(%)	1450 (45.8)	340 (37.7)	515 (45.0)	595 (53.1)	<0.001
SBP, mmHg	110.0 (103.0-120.0)	109.0 (102.0-117.0)	110.0 (103.0-119.0)	112.0 (104.0-123.0)	<0.001
DBP, mmHg	73.0 (67.0-81.0)	72.0 (66.0-78.0)	73.0 (67.0-80.8)	74.0 (68.0-83.0)	<0.001
BMI, kg/m²	27.2 (23.9-32.0)	25.4 (23.0-28.2)	27.3 (23.7-31.6)	29.6 (25.5-35.9)	<0.001
Waist circumstance, cm	87.5 (78.0-98.0)	85.0 (76.0-92.5)	87.0 (77.0-97.5)	91.5 (81.5-103.5)	<0.001
Total physical activity, EU	286.0 (143.0-492.8)	427.0 (256.0-648.0)	286.0 (148.0-479.0)	186.0 (87.0-348.0)	<0.001
Smoking, n(%)	665 (21.0)	89 (9.9)	248 (21.7)	328 (29.3)	<0.001
Drinking, n(%)	2518 (79.5)	690 (76.4)	929 (81.2)	899 (80.2)	0.022
Fasting glucose, mg/dL	83.0 (78.0-90.0)	82.0 (77.0-88.0)	83.0 (78.0-90.0)	84.0 (78.0-91.5)	<0.001
Fasting insulin, uU/mL	12.0 (8.0-17.0)	10.0 (8.0-13.0)	11.0 (8.0-16.0)	14.0 (10.0-20.0)	<0.001

TC, mg/dL	181.0 (160.0-204.8)	178.0 (157.0-200.0)	181.0 (160.0-204.0)	184.0 (162.0-210.5)	<0.001
TG, mg/dL	82.0 (59.0-119.0)	76.0 (55.0-108.0)	80.0 (58.0-119.0)	88.0 (64.0-129.0)	<0.001
HDL-c, mg/dL	49.0 (40.0-59.0)	49.0 (40.0-60.0)	49.0 (40.0-59.0)	48.0 (40.0-59.0)	0.814
LDL-c, mg/dL	110.0 (91.0-133.0)	109.0 (89.0-129.0)	110.0 (90.0-113.0)	112.0 (92.0-123.0)	0.021
VLDL, mg/dL	16.0 (12.0-24.0)	15.0 (11.0-22.0)	16.0 (12.0-24.0)	18.0 (13.0-26.0)	<0.001
Cr, mg/dL	1.0 (0.9-1.1)	1.0 (0.9-1.2)	1.0 (0.9-1.1)	0.9 (0.8-1.0)	<0.001
CRP, ug/ml	1.1 (0.8-2.2)	1.0 (0.8-1.5)	1.1 (0.8-1.9)	1.5 (0.9-3.2)	<0.001
Hypertension, n(%)	483 (15.2)	25 (2.8)	136 (11.9)	322 (28.7)	<0.001
Diabetes, n(%)	166 (5.2)	4 (0.4)	32 (2.8)	130 (11.6)	<0.001
Hyperlipidemia, n(%)	548 (17.3)	41 (4.5)	209 (18.3)	298 (26.6)	<0.001
Medication for hypertension, n(%)	215 (6.8)	7 (0.8)	45 (3.9)	163 (14.5)	<0.001
Lipid-lowering therapy, n(%)	62 (2.0)	0 (0)	26 (2.3)	36 (3.2)	<0.001
Aspirin, n(%)	172 (5.4)	25 (2.8)	62 (5.4)	85 (7.6)	<0.001

Table S4. Participant Characteristics by frailty status.

	All participants (n=3671)	Included participants (n=3168)	No included participants (n=503)	P value
HOMA-IR	2.4 (1.7-3.7)	2.4 (1.7-3.6)	2.8 (1.9-4.8)	<0.001
Age, years	41.0 (37.0-43.0)	41.0 (37.0-43.0)	41.0 (37.0-44.0)	0.898
Women, n(%)	2051 (55.9)	1750 (55.2)	301 (59.8)	0.054
Black, n(%)	1730 (47.1)	1450 (45.8)	280 (55.7)	<0.001
SBP, mmHg	111.0 (103.0-121.0)	110.0 (103.0-120.0)	114.0 (104.0-125.5)	<0.001
DBP, mmHg	73.0 (67.0-81.0)	73.0 (67.0-81.0)	74.0 (67.0-84.0)	0.034
BMI, kg/m²	27.3 (23.9-32.1)	27.2 (23.9-32.0)	28.3 (24.2-33.2)	0.008
Waist circumstance, cm	88.0 (78.5-98.5)	87.5 (78.0-98.0)	91.0 (80.0-102.0)	<0.001
Total physical activity, EU	279.0 (138.0-448.0)	286.0 (143.0-492.8)	244.5 (130.3-454.8)	0.022
Smoking, n(%)	807 (22.0)	665 (21.0)	142 (28.6)	<0.001
Drinking, n(%)	2898 (79.1)	2518 (79.5)	380 (76.8)	0.167
Fasting glucose, mg/dL	83.0 (78.0-90.0)	83.0 (78.0-90.0)	85.0 (78.0-93.0)	0.006
Fasting insulin, uU/mL	12.0 (8.0-17.0)	12.0 (8.0-17.0)	13.0 (9.0-21.0)	<0.001
TC, mg/dL	182.0 (160.0-206.0)	181.0 (160.0-204.8)	187.0 (163.0-212.0)	0.004

TG, mg/dL	83.0 (59.0-122.0)	82.0 (59.0-119.0)	96.0 (64.0-145.0)	<0.001
HDL-c, mg/dL	49.0 (40.0-59.0)	49.0 (40.0-59.0)	48.0 (38.0-59.8)	0.069
LDL-c, mg/dL	110.0 (91.0-133.0)	110.0 (91.0-133.0)	110.0 (91.0-136.0)	0.661
VLDL, mg/dL	16.0 (12.0-24.0)	16.0 (12.0-24.0)	18.0 (12.0-25.0)	0.019
Cr, mg/dL	1.0 (0.9-1.1)	1.0 (0.9-1.1)	1.0 (0.8-1.1)	0.319
CRP, ug/ml	1.2 (0.9-2.3)	1.1 (0.8-2.2)	1.3 (0.9-2.6)	0.002
Hypertension, n(%)	608 (16.7)	483 (15.2)	125 (26.7)	<0.001
Diabetes, n(%)	211 (5.8)	166 (5.2)	45 (9.5)	<0.001
Hyperlipidemia, n(%)	636 (17.8)	548 (17.3)	88 (21.5)	<0.001
Medication for hypertension, n(%)	292 (8.0)	215 (6.8)	77 (15.3)	0.036
Lipid-lowering therapy, n(%)	87 (2.4)	62 (2.0)	25 (5.0)	<0.001
Aspirin, n(%)	226 (6.2)	172 (5.4)	54 (10.9)	<0.001

Figure S1. Cumulative incidence of cardiovascular disease (A), coronary heart disease (B), and stroke (C) by HOMA-IR trajectory.

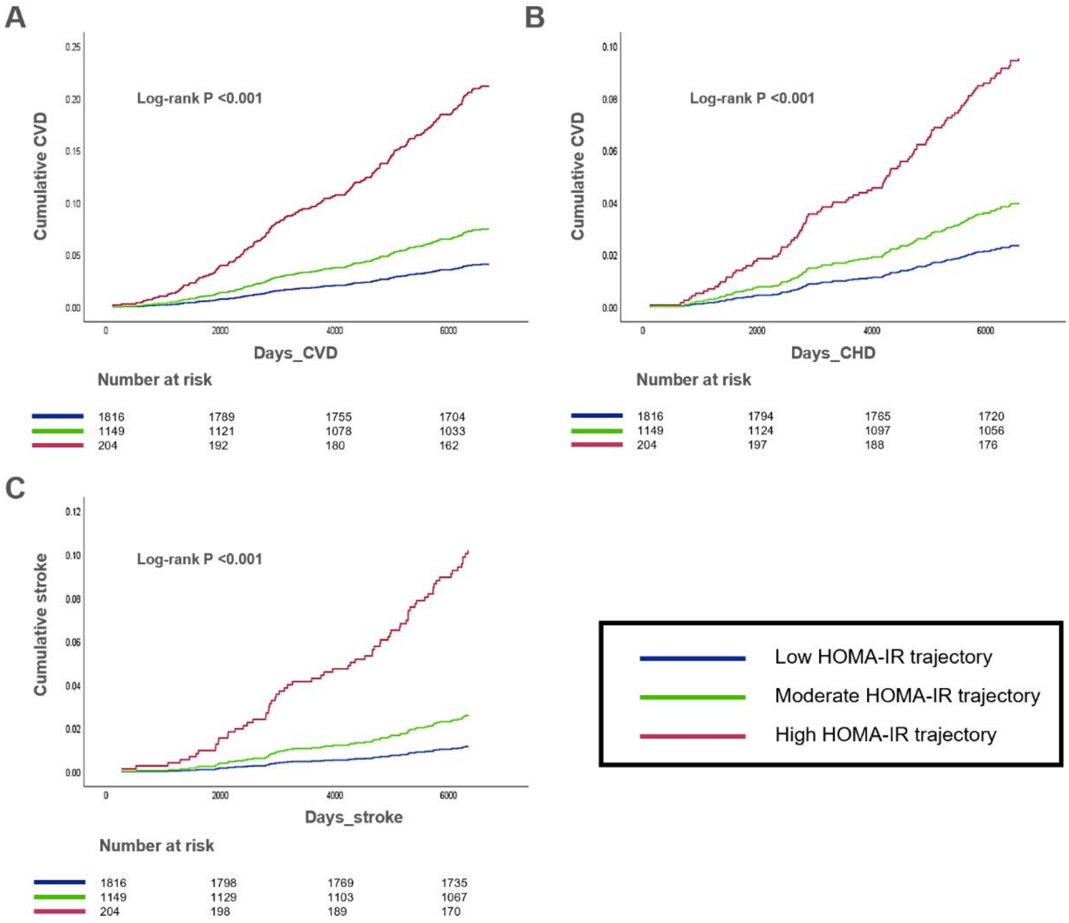

Supplement: Supplementary file 1 — Table S1. The statistics for trajectory models for HOMA‐IR. Table S2. The defects constituting the frailty index. Table S3. Participant Characteristics by frailty status. Table S4. Participant Characteristics by frailty status. Figure S1. Cumulative incidence of cardiovascular disease (A), coronary heart disease (B), and stroke (C) by HOMA‐IR trajectory. [file JCSM-15-1578-s001.pdf]
